# Decoupled Water Electrolysis at High Current Densities Using a Solution-Phase Redox Mediator

**DOI:** 10.1021/acs.energyfuels.5c00092

**Published:** 2025-04-01

**Authors:** Obeten Mbang Eze, Zeliha Ertekin, Mark D. Symes

**Affiliations:** a School of Chemistry, University of Glasgow, Glasgow G12 8QQ, United Kingdom; b Department of Chemistry, University of Cross River State, Calabar, Cross River State 540281, Nigeria

## Abstract

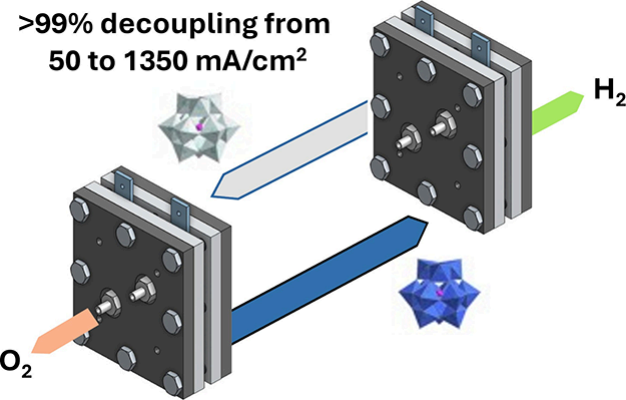

The electrolysis of water using renewably generated power to give “green” hydrogen is a key enabler of the putative hydrogen economy. Conventional electrolysis systems are effective for hydrogen production when steady power inputs are available, but tend to handle intermittent or low-power inputs much less well, in particular because it becomes very difficult to ensure separation of the hydrogen and oxygen products under intermittent or low-power regimes. Decoupled electrolysis offers one potential solution to the problem of interfacing electrolyzers with intermittent and low-power inputs: by allowing the hydrogen and oxygen products of electrolysis to be produced in separate devices to each other, systems in which gas mixtures are inherently much less likely to form can be designed. However, in general, decoupled electrolysis systems operate at rather low current densities (up to a few hundred mA/cm^2^), which detracts somewhat from their suitability for applications. Herein, we constructed a flow system device for decoupled hydrogen production using a solution of the polyoxometalate silicotungstic acid as a liquid-phase decoupling agent. This mediator has been explored as a mediator for decoupled hydrogen evolution before, but in this work, we significantly expanded the range of current densities over which decoupling is demonstrated, from 50 mA/cm^2^ up to 1.35 A/cm^2^, the latter of which exceeds the current densities at which commercial alkaline electrolyzers operate and which begins to approach those achievable with proton exchange membrane electrolyzers. Essentially complete decoupling of the hydrogen and oxygen generation processes is achieved across this full range of current densities, suggesting that rapid oxygen production with coupled redox mediator reduction is possible without compromising on decoupling efficiency.

## 1 Introduction

Hydrogen is a key chemical feedstock and a clean-burning fuel that produces only water when it is consumed.^1^ Today, still around 80% of industrially produced hydrogen comes from steam methane reforming of natural gas, or gasification of coal and petroleum coke.^2^ However, these hydrocarbon resources are nonrenewable and their use for hydrogen production generates substantial amounts of CO_2_ as a byproduct, undermining the sustainability of the hydrogen produced by these methods.^3^ Water electrolysis has the potential to generate hydrogen without generating CO_2_ emissions (so-called “green hydrogen”) if the process is powered by renewably generated electricity. In addition, water electrolysis has the advantage of producing extremely pure zero-carbon hydrogen (>99.9%), ideal for many value-added processes.^4^

In conventional water electrolysis, the water oxidation and reduction reactions are tightly coupled in terms of time, rate and space, as they occur simultaneously at the two electrodes (the anode and cathode) inside the same cell. This coupling introduces operational challenges, such as H_2_/O_2_ crossover,^5^ which is especially problematic when operating under low and/or fluctuating power inputs (such as often characterize renewable power).^6,7^ Such crossover of hydrogen into the oxygen stream accelerates the degradation of expensive components in the electrolyzer^7^ and (if unchecked), can lead to the production of explosive mixtures of hydrogen and oxygen.^8^ Thus, developing more efficient and robust electrolysis systems that are inherently compatible with renewably generated power is vital if green H_2_ production on a large scale is to become a reality.

Decoupled water electrolysis^9,10^ has the potential to solve some of these challenges relating to compatibility with low-power inputs. Decoupled electrolysis works through the use of a redox mediator that allows the production of hydrogen and oxygen to occur at rates that are not intrinsically linked to each other, at different times to each other, and in entirely different reactors to each other. This opens up new possibilities for flexible H_2_ production that are not possible with existing electrolyzers.

In recent years, numerous variations on the concept of decoupled electrolysis involving both soluble and solid-state mediators have since been devised.^11−13^ For example, in 2014 Rausch et al.^14^ showed that decoupled electrolysis using a solution of the polyoxometalate silicotungstic acid as the mediator allows the oxygen-generating step of water splitting to occur electrochemically, while the hydrogen generation step occurs in a completely separate reactor external to the electrochemical cell (“ex-cell”) at rates that are largely independent of the rate of the oxygen generation step. Subsequently, Chisholm et al.^15^ expanded on this study to produce a back-to-back cell design for the oxygen generation and coupled mediator reduction processes, showing that essentially complete decoupling of the oxygen evolution and hydrogen evolution reactions could be achieved with rates of gas crossover that were exceptionally low in comparison to a conventional cell for conventional (“coupled”) electrolysis that was built using the same components. In this previous work, Chisholm et al.^15^ demonstrated that decoupled electrolysis shows substantial promise for minimizing gas crossover across the current density range of 25–500 mA/cm^2^ (and therefore that this system has some in-built flexibility toward low-power inputs), but a number of avenues for optimization and a number of open research questions remain. For example, the flow cell in this previous study employed expensive catalyst-coated membranes, and was only tested at current densities as high as 500 mA/cm^2^. However, conventional proton exchange membrane cells today operate at around 2 A/cm^2^ (or higher).^16^

Herein, a decoupled electrolysis flow system using silicotungstic acid as the redox mediator was assembled, using two electrochemical cells (one for oxygen production and mediator reduction, and the other for hydrogen production and mediator reoxidation). In the oxygen-producing cell, IrO_2_ sprayed directly onto the gas diffusion layer (Ti felt) was employed as the anode catalyst, while in the hydrogen-producing cell 0.5 mg/cm^2^ Pt/C on carbon cloth was employed at the cathode. Hence, neither cell used a catalyst-coated membrane, and instead both used a simpler arrangement of undecorated membrane and catalysts deposited on the gas diffusion layers in contact with the membrane. A range of commercially relevant current densities (0.05 – 1.35 A/cm^2^) was probed as the liquid mediator was circulated continuously between the two cells, showing that the decoupled system can support current densities across this full range while keeping the hydrogen and oxygen generation processes separate.

## 2 Experimental Section

### 2.1 Electrochemical Cell Design and Construction

**Figure 1:**
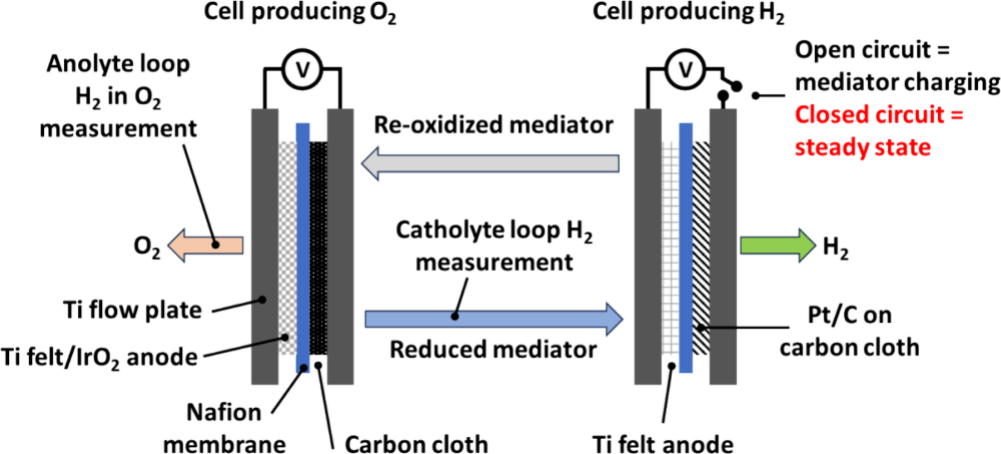
Schematic of the flow cell system designed, constructed, and used in this study.

A schematic of the flow system used in this work is shown in Figure 1. The geometric area of the electrodes in both the oxygen and hydrogen-producing cells was 13.7 cm^2^ (3.7 × 3.7 cm). Silicotungstic acid (H_4_SiW_12_O_40_), purchased from Merck, was used as the redox mediator at a concentration of 0.5 M in ultrapure grade water (15.2 MΩ-cm resistivity).

An exploded view of the oxygen-generating cell used in this study can be found in the Supporting Information (Figure S1). The anode of this cell consisted of a 3 mm-thick titanium serpentine flow field (with flow channels 1 mm deep and 1 mm wide cut into it) and a titanium fiber felt (thickness: 0.3 mm) coated with Ti nanoparticles and loaded with 2.0 mg/cm^2^ IrO_2_ catalyst (supplied by Fuelcellstore) as the active electrode (see below for details of the electrode preparation). These elements were sealed using a 0.127 mm-thick Teflon gasket (supplied by Fuelcellstore). The cathode side also consisted of a titanium serpentine flow field (identical to that used at the anode), and carbon cloth with a microporous layer as the active electrode (Fuelcellstore). No catalyst was added to the cathode side. These elements were sealed using a 0.127 mm-thick Teflon gasket supplied by Fuelcellstore. The anode and cathode were separated by a Nafion 117 membrane supplied by Ion Power. The anode and cathode were compressed against the membrane through 10 mm-thick polytetrafluoroethylene (PTFE) insulating plates and 10 mm-thick titanium end plates, and the bolts fastening the cells were tightened to a torque of 5 N m. Prior to cell assembly, the serpentine flow plates were immersed in phosphoric acid (85%, Thermo Scientific) for 2 h, followed by scrubbing with a sponge and rinsing with deionized water in order to ensure that they were completely clean.

An exploded view of the hydrogen-generating cell used in this study can be found in the Supporting Information (Figure S2). The construction of this cell was similar to that of the oxygen-generating cell. Hence, the anode of this hydrogen-generating cell consisted of a 3 mm-thick titanium serpentine flow plate (identical to those used in the oxygen-generating cell), in combination with a Ti fiber felt (thickness: 3 mm) as the electrode. No catalyst was applied to this Ti fiber felt. The cathode side of this cell consisted of a 3 mm-thick titanium serpentine flow plate in combination with a carbon cloth coated with 0.5 mg/cm^2^ of Pt/C (Fuelcellstore) as the cathode catalyst and transport layer. These elements were sealed using 0.127 mm-thick Teflon gaskets supplied by Fuelcellstore as shown in Figure S2. The anode and cathode were separated by a Nafion 117 membrane supplied by Ion Power. The anode and cathode were compressed against the membrane through 10 mm-thick PTFE insulating plates and 10 mm-thick titanium end plates, and the bolts fastening the cells were tightened to a torque of 5 N m.

### 2.2 Electrode Preparation

The preparation of the anode electrode involved two steps: first, the formation of the Ti microporous layer, and second the coating of this with IrO_2_ (99.9%, Sigma Aldrich) catalyst. In the first step, the Ti fiber felt was modified using 5 μm Ti particles (Alfa Aesar) by weighing out 2.5 g of the Ti particles (≈ 52 mmol) under an inert nitrogen atmosphere into a suitably sized vial in a glovebox (to prevent combustion in air). The vial was sealed with a septum and removed from the glovebox. 2.5 mL of Nafion solution (5 wt % in lower aliphatic alcohols and water, containing 15–20% water, Sigma-Aldrich CAS:31175–20–9) was added to the vial containing the Ti particles using a syringe. The septum was then removed and 10 mL isopropanol was pipetted into the suspension. The vial containing the suspension was then placed into an ultrasonication bath (Fisher Scientific FB 15050) at 37 kHz for 15 min, after which another 10 mL of ethylene glycol (Alfa Aesar, 99%) was added to the solution, and then ultrasonication was continued for a further hour. After this time, the vial containing the suspension was moved to a refrigerator at 4 °C and allowed to cool to 4 °C. Once at 4 °C, spraying was undertaken. To this end, the gas diffusion layer (13.7 cm^2^ Ti fiber felt) was rinsed with water, and ultrasonicated in acetone for 5 min, then allowed to dry. The Ti felt was then sprayed using an AB-182 (Everything Airbrush) double action suction airbrush (0.5 mm), with a 22 mL detachable glass jar bottle attached to the air compressor (Royal max TC-80T single-piston compressor). The spraying support was made of a 5 mm foamboard (see Figure S3 in the Supporting Information). The gas diffusion layer was tightly fitted into the supporting foamboard and held perpendicular to the spraying platform in the fume hood at a comfortable height. After spraying, the gas diffusion layer was then dried in an oven at 100 °C for 5 min and then weighed. In the second step, the IrO_2_ catalyst ink was prepared by mixing 63 mg of carbon black, 55 μL of Nafion solution, and 205 mg of IrO_2_ in a 22 mL airbrush paint bottle. Then, 4.5 mL of isopropanol was added and the mixture was stirred gently with a glass pipette to disperse any clumps. The bottle was sealed with tape to close all openings, then covered with aluminium foil and parafilm. It was then sonicated in an ultrasonic bath with ice for about 3 h. Ice was added every 10–15 min to minimize any temperature increase during the sonication process. After 1.5 h, the bottle was taken out, unsealed, and mixed with a pipette tip to break up any clumps. It was then resealed and returned to the ultrasonic bath for another 1.5 h. After the sonication was complete, the ink was stirred and then stored in the refrigerator at 4 °C until required for spraying. Once at 4 °C, spraying was undertaken. Immediately before spraying, the already prepared and chilled ink was removed from the refrigerator and ultrasonicated at 37 kHz for 30 min on ice, shaken very well, and then returned to the fridge for another 10 – 15 min. The ink was then loaded into the airbrush and one side of the Ti fiber felt was then sprayed inside the fume hood (at a working distance of between 10 and 15 cm), ensuring that the gas diffusion layer was coated evenly by spraying, starting from the perimeter and moving toward the center at a pressure of 1.5 bar. After spraying, the Ti fiber felt was removed from the support and put in an oven in air at 100 °C for 5 min. After this time, the gas diffusion layer was weighed, and further rounds of spraying were undertaken as necessary until the desired loading mass (2.0 mg/cm^2^ of IrO_2_) was obtained.

### 2.3 Electrochemical Experiments

#### 2.3.1 Cyclic Voltammetry

For the cyclic voltammetry tests, a conventional three-electrode system was used, consisting of a glassy carbon button working electrode (area = 0.071 cm^2^), a platinum wire as the counter electrode, and a Ag/AgCl (3 M NaCl) reference electrode. The electrolyte was 0.5 M H_4_SiW_12_O_40_ in ultrapure water. A single chamber cell was used. The cyclic voltammetry measurements were carried out using a Gamry potentiostat version 7.4.8 at a scan rate of 10 mV/s. The potentials reported in this work were converted to the normal hydrogen electrode (NHE) scale using the following equation: E_NHE_ = E_Ag/AgCl_ + E^0^_Ag/AgCl_ + 0.059 pH where E_Ag/AgCl_ is the observed potential during the experiments using a Ag/AgCl (3 M NaCl) reference electrode and E^0^_Ag/AgCl_ is the potential of Ag/AgCl (0.1976 V) versus the normal hydrogen electrode.

#### 2.3.2 Controlled Current Electrolysis

Controlled current electrolysis was performed using a BioLogic SP-150 potentiostat coupled to a BioLogic VMP-3B 20A/20 V booster. Before each experiment, the mediator solution was bubbled with argon for 45 min, to remove any oxygen from the system. The Biologic SP-150 was used to apply a fixed current to the cell making oxygen. The second cell responsible for making hydrogen was driven by an Admiral SquidstatPlus potentiostat to supply the current. The same current was applied across both cells during the working period in order to maintain the system in steady state.

### 2.4 Gas Chromatography

To determine the composition of gases in the anolyte and catholyte loops, 250 μL samples of gas were collected from the headspace of the anolyte and catholyte reservoirs at regular intervals. Before the gas collection from the headspaces, the cell producing hydrogen was purged with argon for 30 min at a flow rate of 250 mL min^–1^. The collected gas samples were then analyzed using a gas chromatography system (Agilent 8860) outfitted with a thermal conductivity detector. This system was configured with two porapak Q columns and a molesieve 13X column. The initial oven temperature during analysis was set to 50 °C, which was held for 4 min, followed by a temperature ramp of 10 °C per minute until reaching 120 °C. The total analysis time was 11 min. Before analysis, the GC system was calibrated using certified hydrogen gas standards (5%, 3%, 2%, and 1% H_2_ in Ar) supplied by CK Gas Products Limited (UK). Linear fits were then produced, allowing the conversion of peak areas into % of H_2_ in the measured gas. From this, the number of moles of hydrogen produced in a given experiment was then calculated by taking into consideration that the volume of 1 mol of an ideal gas at room temperature and pressure is 24 L. This in turn allowed the decoupling efficiency of the mediator reduction step to be calculated by the equation:
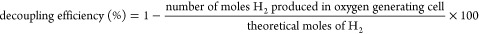
where “theoretical moles of H_2_” corresponds to the amount of hydrogen that one would normally expect to observe in a conventional (“coupled”) electrolyzer, based on the total charge passed.

### 2.5 Physicochemical Characterization

X-ray photoelectron spectroscopy was collected using a Kratos Axis Supra^+^ instrument coupled with monochromated Kα lines of Al (1486.6 eV, 30 mA) as an X-ray source. Data analysis was performed by ESCApe software, employing Gaussian–Lorentzian fitting for each component peak. Scanning electron microscopy (SEM) and X-ray diffractometry (XRD) were performed to observe the surface morphology of the prepared IrO_2_/Ti electrodes as well as to observe the crystallinity of the IrO_2_ layer. The prepared IrO_2_ electrodes were characterized using a Rigaku Mini Flex instrument employing Cu Kα radiation. The scanning diffraction angle 2θ ranged from 10–90° at a speed of 5 min per data point and a scanning rate of 1° min^–1^. SEM (Tescan Clara) equipped with EDX (Oxford Instruments Ultim Max) analysis was employed for probing surface morphology as well as phase composition to confirm the loading mass on the electrode surface. The possible presence of metallic residuals in both the anolyte and catholyte streams following controlled current electrolysis was probed by sampling the electrolyte reservoirs, and then analyzing these samples using inductively coupled plasma optical emission spectroscopy (ICP-OES, Agilent 5900). The residuals were diluted in 2% HNO_3_ before analysis.

## 3 Results and Discussion

### 3.1 Cyclic Voltammetry

**Figure 2:**
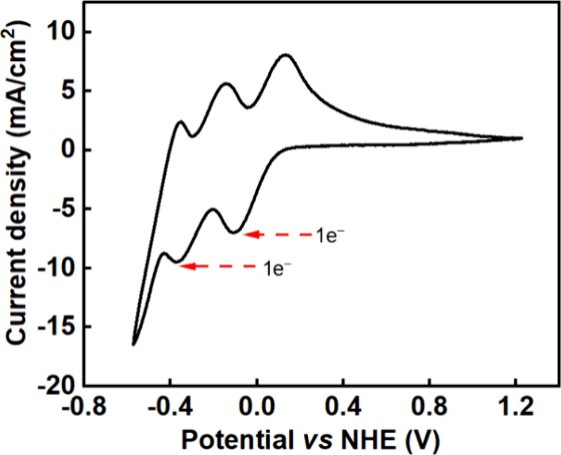
Cyclic voltammogram of silicotungstic acid (0.5 M, pH 0.5) in a conventional three-electrode system at a scan rate of 10 mV/s on a glassy carbon working electrode (0.071 cm^2^), at room temperature (∼25 °C).

Figure 2 shows a cyclic voltammetry study of the redox mediator (0.5 M H_4_SiW_12_O_40_) to determine the position of its redox waves. The first two redox peaks are labeled in Figure 2 and correspond to one-electron processes.^17^ The reduction of H_4_SiW_12_O_40_ to the first reduced state ([H_4_SiW_12_O_40_]^−^, hereafter referred to as the protonated form, H_5_SiW_12_O_40_) has a midpoint at around 0 V vs NHE, while the reduction of H_5_SiW_12_O_40_ to the doubly reduced form [H_4_SiW_12_O_40_]^2–^ (hereafter referred to as H_6_SiW_12_O_40_) has a midpoint at around – 0.3 V vs NHE at pH 0.5. The ratio of the peak currents for the oxidation and reduction processes is unity in both cases, suggesting that these are both reversible electron transfer processes.

Hence, the reduction of H_4_SiW_12_O_40_ to the two electron-reduced form, H_6_SiW_12_O_40_, takes place in two steps, via the formation of the singly reduced species H_5_SiW_12_O_40_. In previous work, Rausch et al.^14^ showed that the introduction of suitable catalysts (e.g., Pt/C) to H_6_SiW_12_O_40_ results in spontaneous hydrogen generation, enabling complete conversion to H_5_SiW_12_O_40_, and converting a further 30% of the H_5_SiW_12_O_40_ onward to the fully oxidized H_4_SiW_12_O_40_ form. Meanwhile, in the presence of Pt/C and hydrogen gas, H_5_SiW_12_O_40_ and the fully oxidized H_4_SiW_12_O_40_ form are in equilibrium.^14^ Importantly, the redox waves for both the H_5_SiW_12_O_40_/H_6_SiW_12_O_40_ couple and the H_4_SiW_12_O_40_/H_5_SiW_12_O_40_ couple lie in between the hydrogen evolution and oxygen evolution potentials under these conditions on a glassy carbon electrode (see Figure S4), enabling the mediator to be fully reduced by two electrons without any competing hydrogen evolution.

### 3.2 Controlled Current Decoupled Electrolysis

**Figure 3:**
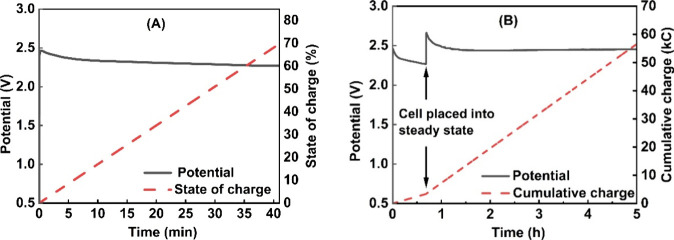
(A) Voltage–time curve showing charging of a 0.5 M H_4_SiW_12_O_40_ solution to 70% state-of-charge at a current density of 100 mA/cm^2^ in the flow system. (B) Steady-state operation curves (at 250 mA/cm^2^) for 0.5 M silicotungstic acid in the flow system. The silicotungstic acid was first charged to 70% state-of-charge at a current density of 100 mA/cm^2^ (see also panel (A)), after which the current density was changed to 250 mA/cm^2^ at around 41 min.

Next, a two-cell flow system (as per Figure 1) was assembled, with 100 mL aqueous 0.5 M fully oxidized silicotungstic acid (H_4_SiW_12_O_40_) solution in both the anolyte and catholyte loops. The flow rate of the anolyte solution through the anode side of the oxygen-generating cell was 40 mL min^–1^, while the flow rate of the catholyte solution through the cathode side of the oxygen-generating cell was set to 250 mL min^–1^. The temperatures of the reservoirs for both the anolyte and catholyte were maintained using oil baths such that the temperatures of both feeds as they entered the cell were 40 °C. Various current densities were then applied to across this oxygen-generating cell (see Table 1) while the circuit on the second (hydrogen-producing) cell was open. The effect of this was to progressively charge the mediator up to a state of “70% charge” (corresponding to the equilibrium position between the H_5_SiW_12_O_40_ and H_4_SiW_12_O_40_ forms when in contact with a Pt/C catalyst at 1 bar as determined by Rausch et al.^14^ i.e., the minimum level of reduction at which one could expect spontaneous hydrogen evolution upon exposure to Pt/C at 1 bar). Given that there was 100 mL of 0.5 M mediator in the catholyte loop, this required the passage of 3370 C across the oxygen-generating cell. Figure 3a shows this charging process for a 0.5 M H_4_SiW_12_O_40_ solution at a current density of 100 mA/cm^2^.

**Table 1:** Average Cell Voltages (over Approximately 5 h) at Different Steady State Current Densities with a Silicotungstic Acid Concentration of 0.5 M, at 40 °C, with an Anolyte Flow Rate of 40 mL min^–1^ and a Catholyte Flow Rate of 250 mL min^–1^^a^

**Steady state current density** (A/cm^2^)	0.05	0.1	0.25	0.5	1.0	1.2	1.35
**Initial state of charge (%)**	70	70	70	70	70	70	70
**Final state of charge (%)**	70	70	70	70	70	70	70
**Total charge passed in steady state (C)**	11363	22646	53225	107543	212672	255450	287385
**Cell Voltages (V)**	2.16	2.25	2.43	2.72	2.94	3.14	3.44
**Decoupling Efficiency (%)**	100	100	99.9	99.9	99.9	99.9	99.9
**Energy Efficiency (%)**	69	66	61	54	50	47	43

a In all cases, the silicotungstic acid mediator solution was charged to 70% state-of-charge by passing 3370 C at a current density of 100 mA/cm^2^ prior to placing the cell into steady state. The energy efficiency was calculated by dividing the thermoneutral voltage for water splitting at 40 °C (∼1.48 V) by the applied cell voltage at each current density.

Once at 70% state-of-charge, the system was then placed into steady state by closing the circuit on the hydrogen-producing cell and setting the current across that cell to be the same as the current across the oxygen-generation cell. In this way, it was possible to reduce the mediator in the oxygen-generation cell and reoxidize it in the hydrogen-producing cell without changing the global state-of-charge of the mediator solution.

Table 1 shows the voltages that needed to be applied across the oxygen-generating cell in order to maintain the system in a steady state at a range of current densities. In all cases, these cell voltages are averaged over an approximately 5 h time period, and in all cases, the mediator solution in the catholyte loop had previously been charged to 70% state-of-charge by applying a current density of 100 mA/cm^2^ before placing the cell into steady state. Figure 3b shows an example voltage–time curve (at a steady state current density of 250 mA/cm^2^), with 53,225 C being passed after placing the system into steady state. Given that the charge required to fully convert this amount of the H_4_SiW_12_O_40_ form of the mediator to the H_5_SiW_12_O_40_ form is 4824 C, 53,225 C corresponds to over 11 turnovers of the mediator, suggesting that the mediator is capable of cycling between the H_5_SiW_12_O_40_ and H_4_SiW_12_O_40_ forms multiple times without noticeable loss of performance on this time scale.

**Figure 4:**
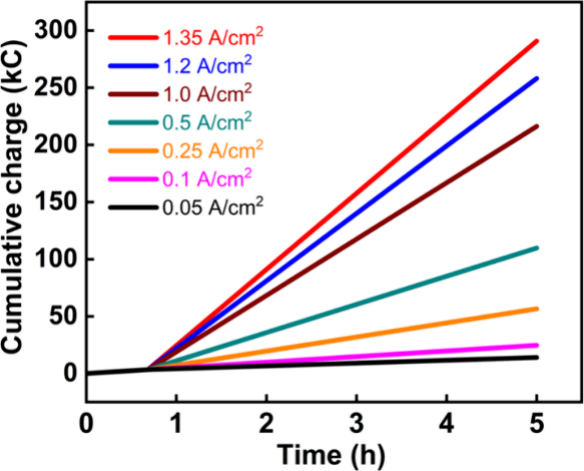
Voltage–time curves at different steady state current densities for 0.5 M silicotungstic acid at 40 °C and a catholyte flow rate of 250 mL min^–1^. In all cases, the silicotungstic acid mediator solution was charged to 70% state-of-charge by passing 3370 C at a current density of 100 mA/cm^2^ prior to placing the cell into a steady state.

Figure 4 shows voltage–time curves for the range of steady-state current densities given in Table 1. During these steady-state experiments, regular gas measurements were taken to ascertain the extent of any hydrogen gas evolution at the cathode of the oxygen-producing cell and the extent to which any hydrogen so produced crossed over into the oxygen stream in the anolyte loop. Table 2 shows the percentage (%) of hydrogen detected in the anolyte and catholyte loops as a function of current density, as well as the decoupling efficiency for mediator reduction calculated on the basis of the amount of hydrogen observed in the catholyte loop (see Figure S5 in the Supporting Information for chromatograms obtained at each current density during a 5 h test period). The percentage of hydrogen in the anode stream (which comes about due to crossover of parasitically generated hydrogen from the cathode to the anode side), reaches a maximum of 0.64% at the highest current density probed here (1.35 A/cm^2^), which is still over six times less than the lower explosion limit of hydrogen in oxygen. Meanwhile, Table 2 shows that at lower current densities (even as low as 0.05 A/cm^2^), the crossover of hydrogen into the oxygen stream at the anode of the oxygen-producing cell was negligible.

**Table 2:** Percent Hydrogen by Volume in the Catholyte and Anolyte Loop Headspaces (Each of Which is 250 mL) after Roughly 5 h of Electrolysis at the Current Densities Indicated, Using a Silicotungstic Acid Concentration of 0.5 M at 40 °C with an Anolyte Flow Rate of 40 mL min^–1^ and a Catholyte Flow Rate of 250 mL min^–1^

**Current density** (A/cm^2^)	% hydrogen in the catholyte headspace	**Decoupling efficiency (%)**	% hydrogen in the anolyte headspace
**0.05**	0.02	99.99+	0.0
**0.1**	0.02	99.99+	0.01
**0.25**	0.03	99.99+	0.02
**0.5**	0.04	99.99+	0.02
**1.0**	0.06	99.99+	0.03
**1.2**	0.74	99.99	0.05
**1.35**	0.83	99.99	0.64

After running in steady state for around 5 h, the mediator solution in the catholyte loop was fully reoxidized by opening the circuit on the oxygen-generating cell and applying a potential to the hydrogen-generating cell only. The amount of charge needed to completely reoxidize the mediator was measured and compared to the amount of charge used to reduce the mediator initially. The ratio of these two values then defines the “final state-of-charge” in Table 1, with a value of 70% implying that the state-of-charge at the end of the steady state experiment was the same as the state-of-charge at the beginning (i.e., that the average charge of the mediator remained at essentially 3:7 H_4_SiW_12_O_40_:H_5_SiW_12_O_40_ throughout).

### 3.3 Electrode Characterization

**Figure 5:**
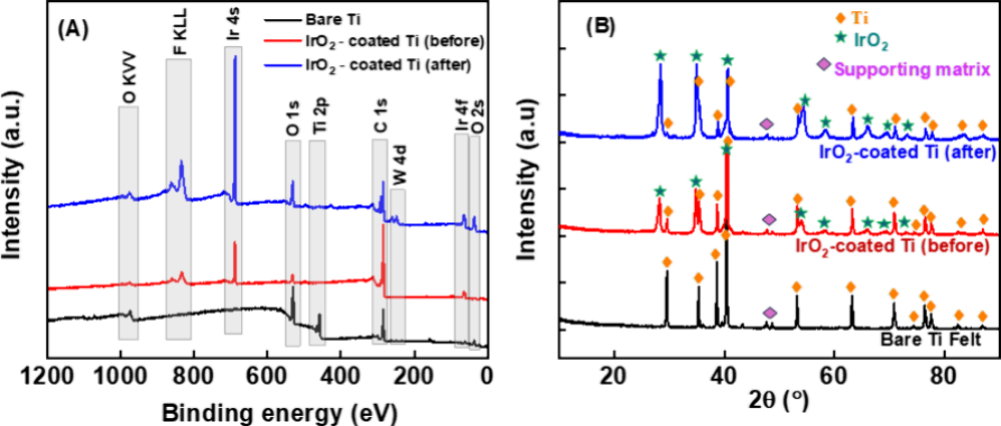
Wide-scan (A) XPS and (B) XRD spectra of a bare Ti felt and an IrO_2_ coated Ti electrode before and after electrolysis in a flow cell containing 0.5 M silicotungstic acid at 40 °C and an anolyte flow rate of 40 mL min^–1^.

The surface of the IrO_2_/Ti felt electrode was analyzed by X-ray photoelectron spectroscopy (XPS) before and after conducting electrolysis at different current densities at an anolyte flow rate of 40 mL min^–1^ for 5 h at 40 °C. Figure 5a illustrates the XPS survey spectra for the prepared IrO_2_/Ti felt electrodes before and after electrolysis, alongside a bare (undecorated) Ti felt for comparison. “After electrolysis” here means after extensive usage at a range of current densities between 0.05 and 1.35 A/cm^2^. The tungstic (W) and fluoro (F) peaks were attributed to silicotungstic acid and Nafion (the latter was used as an ionomer during electrode preparation) respectively. The XPS analysis for the bare Ti substrate can be found in the Supporting Information (Figure S6). Deconvolution of the XPS spectra was performed to discern specific binding energies (eV) for the Ti 2p, O 1s, and Ir 4f signals at the surface of the IrO_2_ coated electrode, both pre- and post electrolysis (Figure S7).

After 180 days of contact with the silicotungstic acid in the cell (with electrolysis at varying current densities across the range 0.05–1.35 A/cm^2^), analysis of the Ti 2p peaks at 459.3 eV (Ti 2p_3/2_) and 465.0 eV (Ti 2p_1/2_) indicated some breakthrough of the underlying Ti, but not total loss of the Ir catalyst as Ir is still clearly found at the surface of the electrode (Figure S7b) after electrolysis.^18^ The O 1s XPS spectra of the IrO_2_-coated electrode before and after the experiment can be deconvoluted into three peaks (Figure S7c,d). Slight differences are observed. The primary O 1s peak at 529.8 eV is attributed to Ir–O bonding, while a minor peak at 532.9 eV corresponds to surface hydroxyl groups, which are present on both electrodes. On the other hand, the peak at 528.4 eV, corresponding to 2-fold coordinated bridging oxygen atoms, is evident before electrolysis, whereas the peak around 531.3 eV is related to oxygen vacancies (oxygen defects) that occur after electrolysis.^18−20^ The XPS spectra of Ir 4f (Figure S7e,f), exhibit similarity before and after the experiments. The XPS spectra of Ir 4f revealed distinct peaks at 65.1 eV (4f_5/2_) and 62.1 eV (4f_7/2_), indicating the presence of the Ir^3+^ state. Additionally, peaks were observed at 66.4 eV (4f_5/2_) and 63.5 eV (4f_7/2_), corresponding to the unscreened component of the Ir^4+^ state.^20,21^ Additionally, the peak at 66.4 eV is attributed to the shakeup satellite of the Ir^3+^ species.^19,22,23^

**Figure 6:**
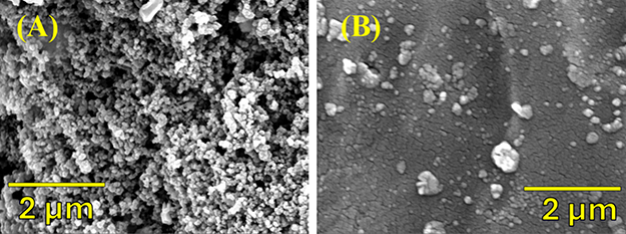
SEM images of (A) the IrO_2_/Ti electrode prepared by air spraying deposition before electrolysis and (B) after electrolysis at a range of current densities in a flow cell containing 0.5 M silicotungstic acid at 40 °C and an anolyte flow rate of 40 mL min^–1^.

To probe the phase compositions of the Ti/IrO_2_ coated electrode, X-ray diffraction (XRD) analysis was employed. The diffractograms revealed characteristic peaks related to the substrate and well-defined peaks for the IrO_2_ layer, characterestic of a crystalline structure (Figure 5b). The XRD data for the main peaks observed in the diffractograms were compared with XRD data from the JCPDS (Joint Committee of Powder Diffraction Standards). As shown in Figure 5b, characteristic peaks corresponding to Ti felt were fairly narrow and strong, which were assigned to the rutile crystal structure. In the case of Ti/IrO_2_, single peaks were observed for the 110 and 220 phases (2θ = 28°, 58°, 65°, and 69° respectively), with PDF card number 1538153, which confirms the presence of IrO_2_ after the electrolysis process. Similar observations have been reported in the literature.^23,24^ Furthermore, diffraction peaks corresponding to the Ti substrate were observed, but no TiO_2_ was detected. SEM micrographs (Figure 6) depict the morphology of the IrO_2_/Ti surface and, combined with EDX analysis (Figure S8), confirm the presence of iridium on the titanium surface.

Figure 6a,b presents the surface morphology of the IrO_2_/Ti electrode. The micrographs and the EDX indicate that before electrolysis the IrO_2_ particles are evenly dispersed on the Ti felt surface, as expected according to the air spraying deposition process, forming large spots and numerous nanoparticles, which are shown in the SEM images as white patches (Figure 6a). One can also notice that the IrO_2_ on the Ti surface (Figure 6b) has not been completely leached out even after electrolysis. Based on EDX analysis the Ir/Ti composite had 4.5 wt % Ti and 39.3 wt % Ir before electrolysis, and 59.0 wt % Ti and 4.7 wt % Ir after electrolysis. The change in the composition of Ir after electrolysis can be attributed to the long contact (>180 days) with the silicotungstic acid solution at a range of different current densities (0.05 – 1.35 A/cm^2^). The composition of other elements present at the electrode is shown in the Supporting Information (Figure S8).

Finally, to determine the extent to which Ir had been leached from the anode after 180 days in contact with the silicotungstic acid solution at a range of different current densities (0.05 – 1.35 A/cm^2^), ICP-OES measurements were performed. The samples were diluted in 2% HNO_3_ and were then measured by ICP-OES. The ICP-OES measurements showed Ir below the detection limit (≤0.1 ppm) indicating no leached Ir in the anolyte stream after a 24 h test period with no leachates found in the cathode stream. However, 576.83 ppm of Ir was found to be leached into the anode stream after 180 days in contact with the silicotungstic acid solution, with 34.45 ppm found in the catholyte stream after this period.

## 4 Conclusions

In this work, we have demonstrated the operation of a flow cell system using silicotungstic acid as a redox mediator for the near-complete decoupling of the oxygen and hydrogen evolution reactions using a water oxidation catalyst (IrO_2_) applied directly onto the gas diffusion layer (Ti felt), across a range of current densities. The system was placed into steady state for numerous turnovers of the mediator at these current densities, turning over around 60 times at the highest current density probed (1.35 A/cm^2^). Across the full range of current densities that were probed (0.05 – 1.35 A/cm^2^), the decoupling efficiency remained well in excess of 99%, suggesting that even at the higher current densities reported here reduction of the mediator can be engineered to out-compete hydrogen evolution inside the oxygen-generating electrochemical cell. Current densities of 1.35 A/cm^2^ begin to approach those obtainable with conventional proton exchange membrane electrolyzers, and highlight that rapid oxygen production with coupled redox mediator reduction is possible without compromising on the decoupling efficiency. At the other end of the scale, current densities as low as 0.05 A/cm^2^ could be harnessed for oxygen production and mediator reduction without any cogeneration of hydrogen, and thus no risk of hydrogen permeation into the oxygen stream.

The main trade-off for this decoupled electrolysis system is in terms of the efficiency of the electrochemical process: as Table 1 shows, the electrochemical step could still benefit from optimization, and (as the mediator is necessarily somewhat harder to reduce than protons), the electrochemical step is inherently less efficient than in a conventional (coupled) electrolyzer. Such inefficiencies may yet be minimized when full balance of plant is considered, but that is beyond the scope of this study. For the electrochemical system in isolation, the footprint of the unit is likely to be larger than for a conventional electrolyzer, as additional storage tanks and pumping would be required.

On the other hand, decoupled electrolysis may allow alternative cell layouts to be considered, which might have benefits for reducing electrochemical cell costs. For example, by moving the catalyst for the oxygen evolution reaction to the gas diffusion layer (as opposed to applying this catalyst directly on the membrane as a membrane electrode assembly), the construction of the cells in this paper was simplified, raising the prospect that membranes of different sorts could be readily swapped in and out of the electrochemical cell. Very little attempt was made to optimize the deposition or performance of these IrO_2_/Ti anodes, but nevertheless they displayed passable activity and stability under the reaction conditions, still retaining sufficient Ir to be active after 180 days in contact with the electrolyte and after having performed many hours of electrolysis. Given the expense of membrane electrode assemblies, an ability to deposit catalysts on the gas diffusion layer and to use undecorated membranes could be a distinct advantage, especially as decoupled systems based on liquid electrolytes such as in this case only require a membrane to prevent the reduced mediator from being reoxidized at the anode (which would result in wasteful redox cycling). Moving the catalyst to the gas diffusion layer should allow a wider range of potential separators to be screened and assessed for efficacy, and work toward these ends is currently underway in our laboratory.

## Data Availability

The data underpinning this study have been deposited in the University of Glasgow’s Enlighten database under accession code 10.5525/gla.researchdata.1929.

## Supporting Information Available

Flow cell components and data from cyclic voltammetry, gas chromatography, X-ray photoelectron spectroscopy, and scanning electron microscope with energy-dispersive X-ray spectroscopy (PDF)
