# Supplementary material for: Decoupled Water Electrolysis at High Current Densities Using a Solution-Phase Redox Mediator
Source: Energy Fuels. 2025 Apr 1;39(14):7129–36. doi: 10.1021/acs.energyfuels.5c00092 (PMC11995369; doi:10.1021/acs.energyfuels.5c00092)
Supplement: Supplementary file 1 — ef5c00092_si_001.pdf [file ef5c00092_si_001.pdf]

Supporting Information for:

Decoupled water electrolysis at high current densities using a solution-phase redox mediator

Obeten Mbang Eze,[a,b] *Zeliha Ertekin*[a], *and Mark D. Symes**[a]

[a]*School of Chemistry, University of Glasgow, Glasgow, G12 8QQ, United Kingdom*
[b]*Department of Chemistry, University of Cross River State, Calabar, Cross River State, Nigeria.*
**Email:* mark.symes@glasgow.ac.uk

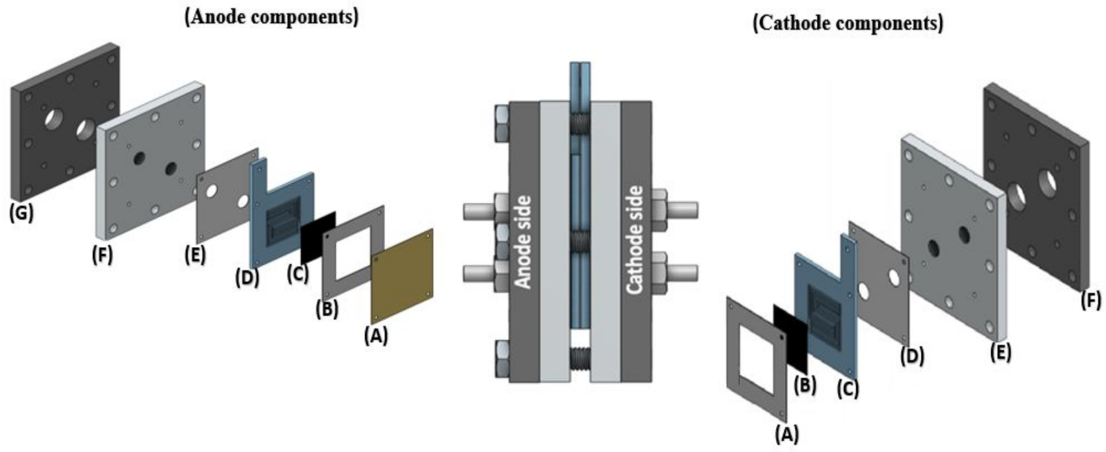

Fig. S1. An exploded view of the components used to assemble the oxygen-producing flow electrochemical cell used in this study. Components are as listed below (these are described in the main text):

Anode-side components

(A) Nafion 117 membrane

(B) Teflon gasket

(C) Ti fiber felt coated with Ti nanoparticles and IrO_2 (gas diffusion layer)

(D) Ti serpentine flow plate

(E) Polytetrafluoroethylene (PTFE) insulating gasket

(F) PTFE insulating plate

(G) Titanium end plate

Cathode-side components

(A) Teflon gasket

(B) Cabon cloth with a microporous layer

(C) Ti serpentine flow plate

(D) Polytetrafluoroethylene (PTFE) insulating gasket

(E) PTFE insulating plate

(F) Titanium end plate

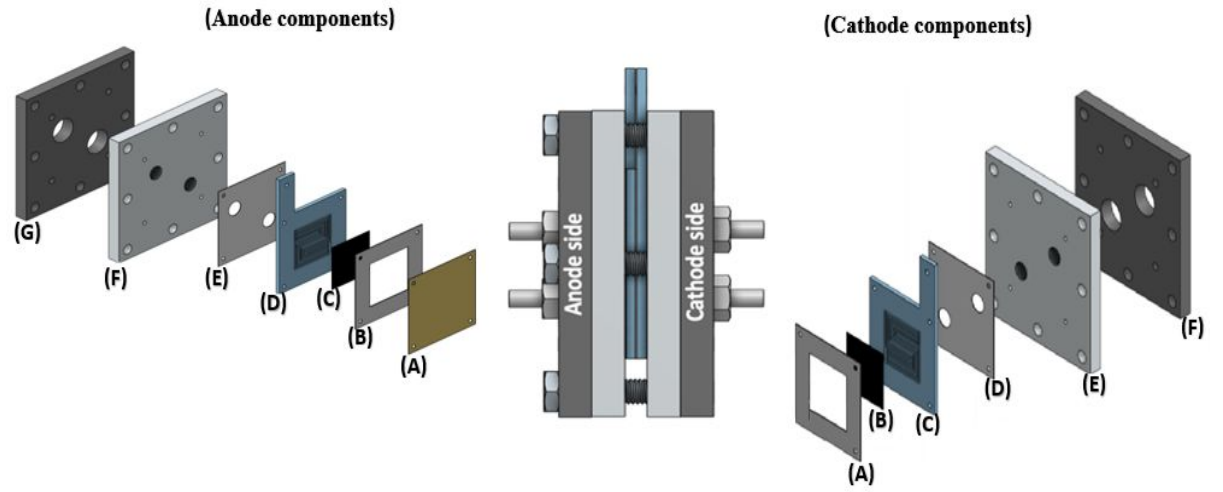

Fig. S2. An exploded view of the components used to assemble the hydrogen-producing flow electrochemical cell used in this study. Components are as listed below (these are described in the main text):

Anode-side components

(A) Nafion 117 membrane

(B) Teflon gasket

(C) Ti fiber felt (no catalyst added)

(D) Ti serpentine flow plate

(E) Polytetrafluoroethylene (PTFE) insulating gasket

(F) PTFE insulating plate

(G) Titanium end plate

Cathode-side components

(A) Teflon gasket

(B) Vulcan carbon cloth with 0.5 mg Pt/C

(C) Ti serpentine flow plate

(D) Polytetrafluoroethylene (PTFE) insulating gasket

(E) PTFE insulating plate

(F) Titanium end plate

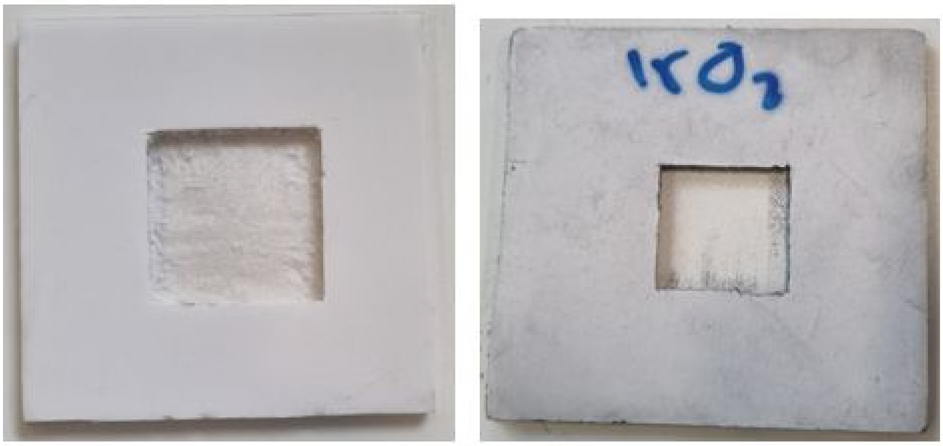

Fig. S3. Foamboard structure used to support the Ti felt electrode during the air-spraying of the IrO_2 catalyst onto the Ti felt, as described in the main text.

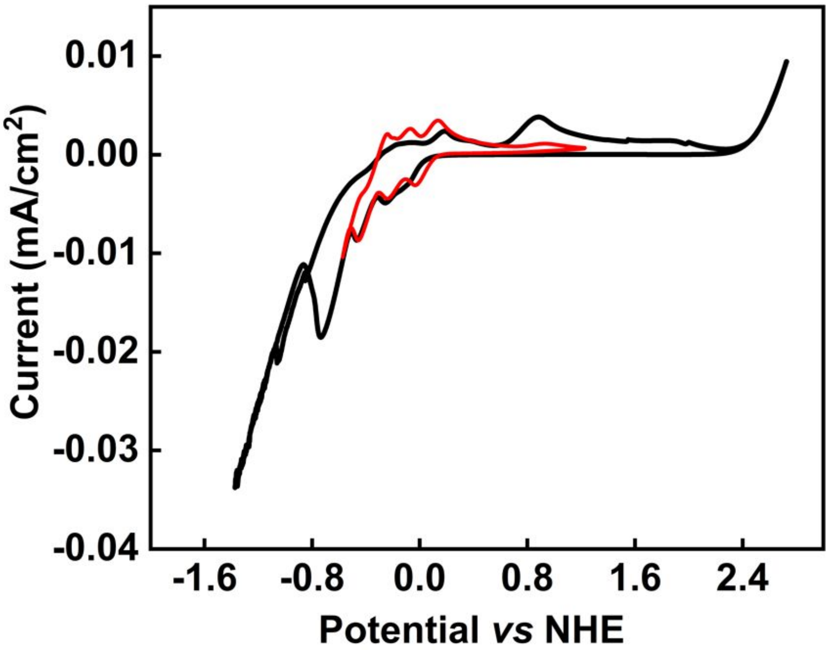

Fig. S4. Restricted potential window cyclic voltammogram of silicotungstic acid (0.5 M, pH 0.5, red line) and an expanded curve over a wider potential range under the same conditions (black line) in a conventional three-electrode system at a scan rate of 10 mV/s on a glassy carbon working electrode (0.071 cm^2), at room temperature (~25 ºC).

Gas chromatography

These chromatograms show the H_2 peak observed at each retention time, along with a small peak for O_2 (from the air) in the catholyte stream (Fig. S5b) and negligible hydrogen in the anolyte stream (Fig. S5a) for most current densities, suggesting excellent decoupling of the hydrogen evolution reaction from the oxygen evolution reaction in the oxygen-generating cell.

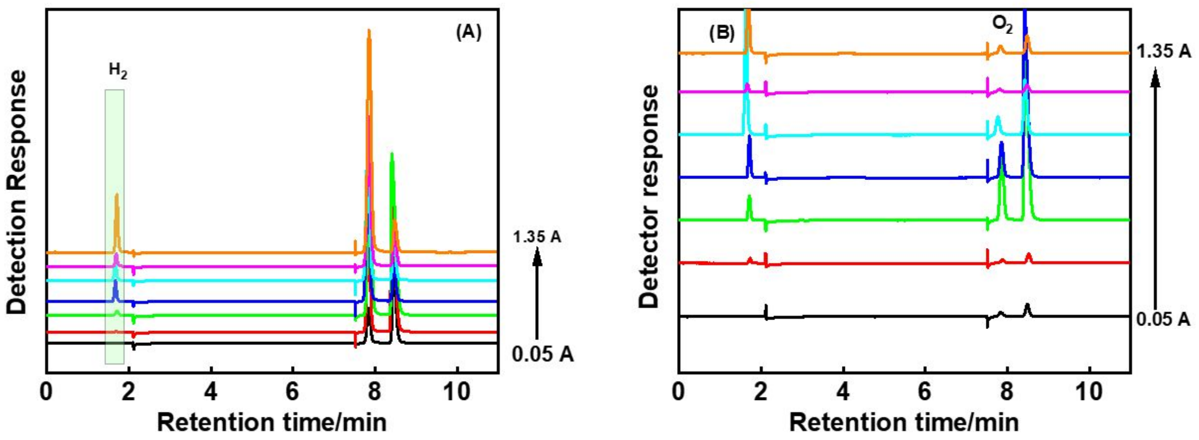

Fig. S5. GC measurements for % of hydrogen present in the anolyte (a) and catholyte stream (b) at different current densities in a flow cell containing 0.5 M silicotungstic acid at 40 °C and a catholyte flow rate of 250 mL min^{-1}. Hydrogen has a retention time of 1.8 minutes, oxygen 7.9 minutes, and nitrogen 8.7 minutes. The nitrogen and some of the oxygen originate from air leaks within the GC apparatus; in panel b, almost all the oxygen present originates from the air.

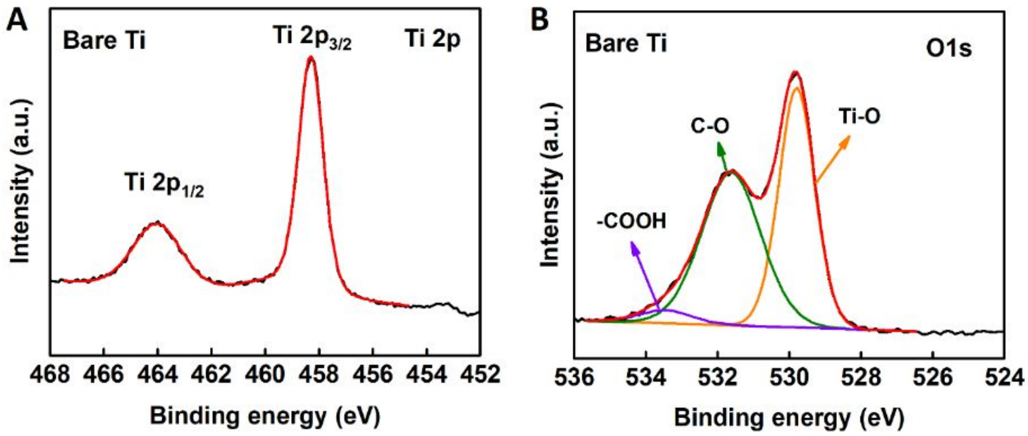

Fig S6. Deconvoluted Ti 2p (a) and O 1s (b) for a fresh (undecorated) Ti felt.

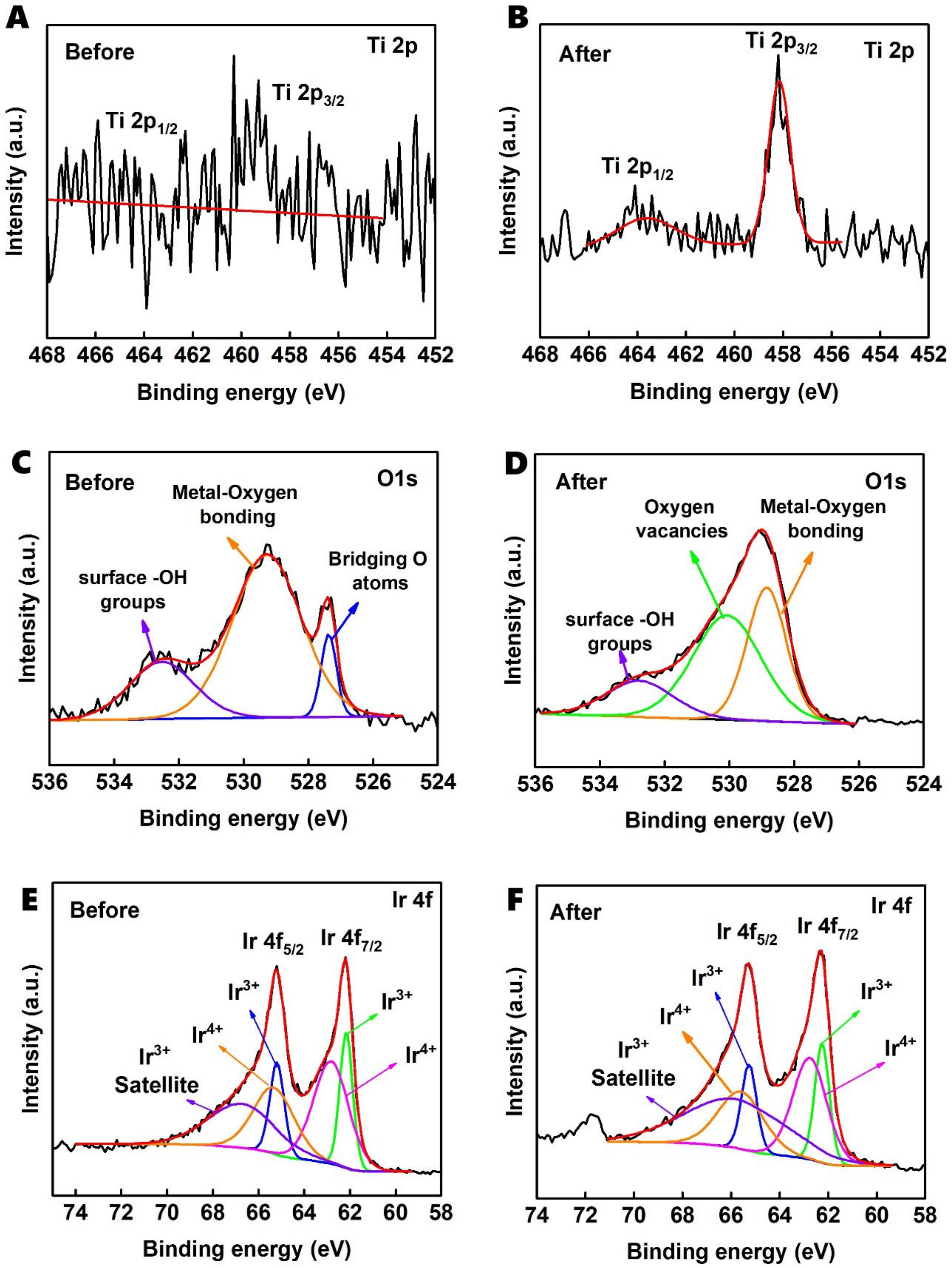

Fig. S7. Deconvoluted XPS spectra for an IrO_2-coated Ti felt electrode as follows: (a) Ti 2p before electrolysis, (b) Ti 2p after electrolysis, (c) O 1s before electrolysis, (d) O 1s after electrolysis, (e) Ir 4f before electrolysis, (f) Ir 4f after electrolysis. Electrolysis was performed in a flow cell containing 0.5 M silicotungstic acid at 40 °C and a catholyte flow rate of 250 mL min^{-1}.

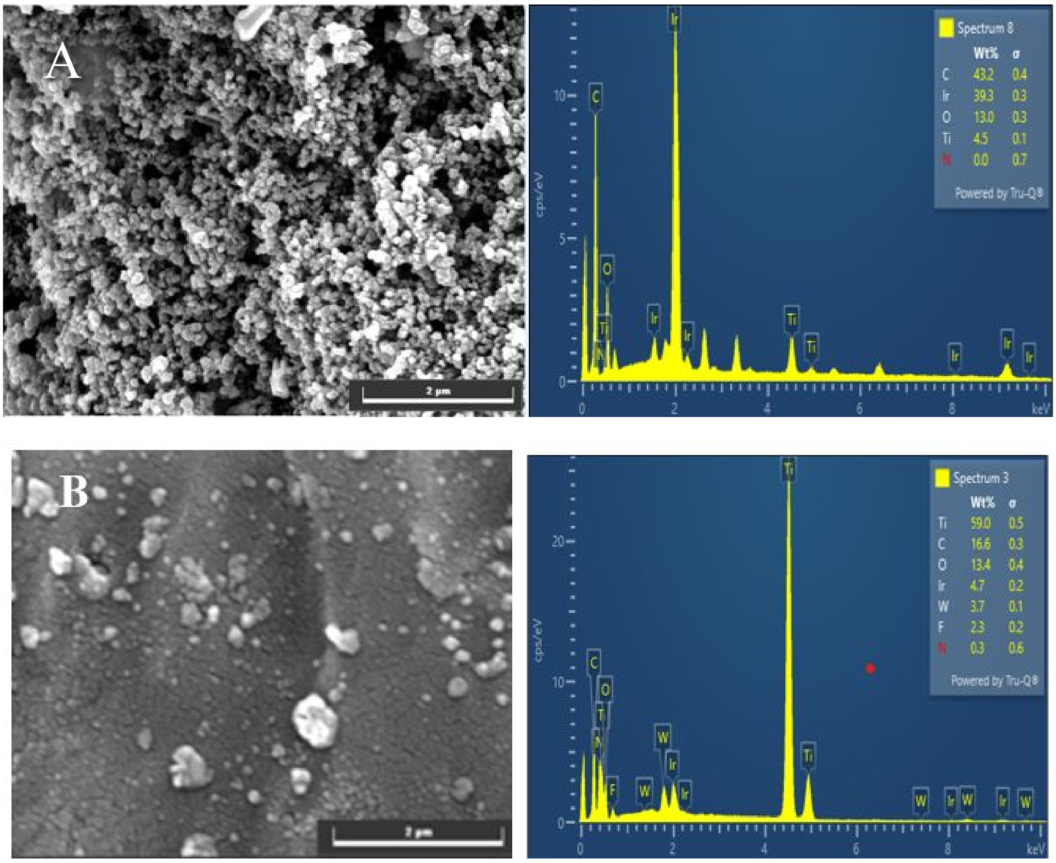

Fig. S8. SEM/EDX images of (**A**) a IrO_2/Ti electrode prepared by air spraying before electrolysis and (**B**) after electrolysis in a flow cell containing 0.5 M silicotungstic acid at 40 °C and a catholyte flow rate of 250 mL min^{-1}.
